# FAD influx enhances neuronal differentiation of human neural stem cells by facilitating nuclear localization of LSD1

**DOI:** 10.1002/2211-5463.12331

**Published:** 2017-10-17

**Authors:** Kazumi Hirano, Masakazu Namihira

**Affiliations:** ^1^ Molecular Neurophysiology Research Group Biomedical Research Institute The National Institute of Advanced Industrial Science and Technology (AIST) Tsukuba Japan

**Keywords:** flavin adenine dinucleotide, lysine‐specific demethylase‐1, neural stem cells

## Abstract

Flavin adenine dinucleotide (FAD), synthesized from riboflavin, is redox cofactor in energy production and plays an important role in cell survival. More recently, riboflavin deficiency has been linked to developmental disorders, but its role in stem cell differentiation remains unclear. Here, we show that FAD treatment, using DMSO as a solvent, enabled an increase in the amount of intracellular FAD and promoted neuronal differentiation of human neural stem cells (NSCs) derived not only from fetal brain, but also from induced pluripotent stem cells. Depression of FAD‐dependent histone demethylase, lysine‐specific demethylase‐1 (LSD1), prevented FAD‐induced neuronal differentiation. Furthermore, FAD influx facilitated nuclear localization of LSD1 and its enzymatic activity. Together, these findings led us to propose that FAD contributes to proper neuronal production from NSCs in the human fetal brain during development.

**Abbreviations**
FAD: flavin adenine dinucleotide
FMN: flavin mononucleotide
hfNSC: human fetal neural stem cell
hiPS‐NSC: human induced pluripotent stem cell‐derived neural stem cell
LSD1: lysine‐specific demethylase‐1
NSC: neural stem cell

Flavin adenine dinucleotide (FAD), synthesized from riboflavin (also known as vitamin B2), is a redox cofactor that ensures the functionality of enzymes, that is, flavoenzymes, that are involved in oxidative metabolism of carbohydrates, amino acids, fatty acids, and in mitochondrial electron transport 1, 2, 3. Interruption of riboflavin ingestion and its metabolism toward FAD induces the depletion of intracellular FAD levels, resulting in reduced energy production 4. Several studies have reported that dietary riboflavin deficiency in human and experimental animal models is linked to tumorigenesis, cardiovascular disease, and various developmental and neurological disorders 4, 5. In addition, a previous study using human fetal brain cells has revealed that the administration of riboflavin to culture medium promotes the growth of these cells 6. Although these reports suggest that FAD and its dependent flavoenzymes are essential for human development, the roles of FAD in the differentiation of tissue‐specific stem cells that are responsible for organ development remain unclear.

In transcriptional regulation, FAD acts as a coenzyme of lysine‐specific demethylase 1 (LSD1, also known as KDM1A), which is transcriptional corepressor catalyzing the demethylation of mono‐ or dimethylation at histone H3 lysine 4 (H3K4me1/2) 7. The repressing effect of LSD1 is exerted by cooperating with its binding partners, including CoREST (RCOR1) 8, histone deacetylase 1 (HDAC1) 9, and REST 10. These LSD1 complexes are responsible for the repression of neuronal genes in non‐neuronal cells 11. Moreover, it has been reported that LSD1 demethylation activity modulates energy metabolism via transcriptional regulation of genes encoding key metabolic factors, such as PPARγ coactivator 1‐α (PGC1α), which is the master regulator of mitochondrial biogenesis 12, 13, and that this metabolic regulation depends on FAD biosynthesis 12. In addition to these reports describing the role of LSD1 in metabolism, several studies using mice that have undergone conditional deletion of *lsd1* in various tissues suggest that LSD1 is indispensable for the maintenance and differentiation of tissue‐specific stem cells 14, 15, 16. Furthermore, it has also been shown that the degradation of LSD1 expression in human embryonic stem cells (ESCs) leads to the aberrant expression of developmentally regulated genes 17. Given these findings, it is reasonable to assume that FAD also participates in the regulation of differentiation of tissue‐specific stem cells during development.

In the mammalian fetal cerebral cortex, neural stem cells (NSCs) self‐renew to increase their numbers 18, 19, 20, and then differentiate into neurons and glial cells 21, 22, 23. In the mouse developing cerebral cortex, interference with LSD1/CoREST results in abnormal neuronal migration and differentiation, because of interruption of the NOTCH pathway 24, 25. Moreover, in our recent study using human fetal NSCs (hfNSCs), disturbance of LSD1 activity by means of its inhibitor caused suppression of neuronal differentiation by regulating the expression of the Hes‐related family bHLH transcription factor with YRPW motif‐like gene (*HEYL*), which is a NOTCH target gene 26, suggesting that LSD1 activity is necessary for neurogenesis in the fetal cerebral cortex. These studies prompted us to examine whether FAD is also involved in neuronal differentiation through the regulation of LSD1 activity.

In this study, we show that FAD treatment using DMSO as a solvent to facilitate membrane permeability enabled an increase in the amount of intracellular FAD and promoted the neuronal differentiation of NSCs. We further found that a FAD influx promoted the nuclear localization of LSD1, and its demethylation activity in these cells. These findings shed light on the novel role of FAD, associated with LSD1 activity, in NSCs during the brain development of the human fetus.

## Materials and methods

### Antibodies

For immunostaining, we used anti‐LSD1 (ab17721; Abcam, Cambridge, MS, USA), anti‐CTIP2 (ab18465; Abcam), anti‐SOX2 (MAB2018; R&D Systems Inc., Minneapolis, MN, USA), anti‐DCX (C‐18; SantaCruz, Dallas, TX, USA), anti‐βIII‐tubulin (MAB1195; R&D Systems Inc.), anti‐GFAP (Z0334; Dako, Santa Clara, CA, USA), and anti‐GFP (GFP‐1010; Aves Labs Inc., Tigard, OR, USA) as the primary antibodies, and CF488/568/647 donkey anti‐rabbit IgG (Biotium Inc., Fremont, CA, USA), CF488/568 donkey anti‐mouse IgG (Biotium Inc.), CF488/568/647 donkey anti‐goat IgG (Biotium Inc.), CF568 donkey anti‐rat IgG (Biotium Inc.), and CF488 donkey anti‐chicken (Biotium Inc.) as the secondary antibodies. For western blotting, we used anti‐H3K4me1 (ab8895; Abcam), anti‐H3K4me2 (ab7766; Abcam), anti‐H3K4me3 (ab8580; Abcam), anti‐histone H3 (ab1791; Abcam), anti‐β‐actin (ab8226; Abcam), anti‐LSD1 (ab17721; Abcam), anti‐lamin B1 (PM064; MBL, Nagoya, Aichi, Japan), and anti‐GAPDH (MAB374; EMD Millipore, Billerica, MA, USA) as the primary antibodies, and anti‐mouse IgG H&L [horseradish peroxidase (HRP)] (ab97023) and anti‐rabbit IgG H&L (HRP; ab97051) as the secondary antibodies.

### Human fetal neural stem cell culture and *in vitro* differentiation

All human cell and tissue experiments were approved by the AIST Instituted Human Experiment Committee and Ethical Committee. hfNSCs, isolated from the human cerebral cortex of a male fetus at embryonic week 14, were purchased from Phoenix Songs Biologicals, Inc. (Branford, CT, USA). hfNSCs were cultured in N2‐supplemented Dulbecco's modified Eagle's medium with F12 (DMEM/F12, GIBCO, Waltham, MA, USA) containing a 0.1% B27 supplement (GIBCO), 10 ng·mL^−1^ human basic fibroblast growth factor (FGF; R&D Systems Inc.), and 20 ng·mL^−1^ human epidermal growth factor (EGF; PeproTech, Inc., Rocky Hill, NJ, USA) on culture dishes that had been precoated with poly‐l‐ornithine (Sigma‐Aldrich, St. Louis, MO, USA) and laminin (Corning, Corning, NY, USA). For neuronal differentiation, hfNSCs were placed into neurobasal medium (GIBCO) containing 2% B27 supplement (GIBCO) and 0.5 mm l‐glutamine (Nacalai Tesque, Inc., Kyoto, Japan). Different concentrations of FAD (Sigma Aldrich), riboflavin (Sigma Aldrich), or flavin mononucleotide (FMN; Sigma Aldrich) were added to examine their influence on differentiation.

For immunostaining, cells were washed with PBS, fixed in 4% paraformaldehyde in PBS, and stained with appropriate antibodies. Before fixation, 2 μg·mL^−1^ propidium iodide (PI) or 10 μm 5‐ethynyl‐2′‐deoxyuridine (EdU) was incorporated into the cultured cells for 10 or 120 min at 37 °C. Nuclei were stained using Hoechst 33342 (Dojindo Laboratories, Kumamoto, Japan). Stained cells were visualized with a fluorescence microscope (BX53; Olympus, Tokyo, Japan).

### Quantitative determination of amount of intracellular FAD

The intracellular FAD amount was measured using an FAD assay kit (ab204719; Abcam) following the product's protocol. In brief, homogenized hfNSCs were deproteinized using trichloroacetic acid (ab204708; Abcam). Samples were incubated with an FAD enzyme mix, OxiRed Probe, and assay buffer for 5–15 min in triplicate before measurement by the fluorometric method (Ex/Em = 535/587 nm) using SpectraMax Gemini (Molecular Devices Inc., Sunnyvale, CA, USA).

### Lentiviral constructs, preparation of lentivirus, and viral infection

The lentivirus vector used to express a short hairpin RNA (pLLX) was generated by Z. Zhou and M.E. Greenberg. pLLX is a dual‐promoter lentivirus vector constructed by inserting the U6 promoter‐driven shRNA cassette 5′ to the ubiquitin C promoter in the FUIGW plasmid 27, 28. pLLX was modified to express GFP together with a puromycin resistance gene under the ubiquitin C promoter. shRNA for the LSD1 lentivirus constructs were generated by inserting oligonucleotides into the *Hpa*I and *Xho*I sites of pLLX. The oligonucleotide target sequence of human *LSD1* is GGAGCTCCTGATTTGACAAAG (*LSD1* KD). For overexpression experiments, full‐length human *LSD1* (NM_015013) was provided by the RIKEN BioResource Center (RIKEN BRC, Ibaraki, Japan) 29, 30, 31, 32 and was cloned into the CSII lentiviral vector (provided from RIKEN BRC, Miyoshi, Japan). To prepare the lentivirus, HEK293T cells were cotransfected with these constructs and lentiviral packaging vectors (pCAG‐HIVgp and pCMV‐VSV‐G‐RSV‐Rev). The medium was changed to hfNSC growth medium at 24 h after transfection. The culture supernatants were collected 48 h after medium replacement and were concentrated using the Lenti‐X Concentrator following the manufacturer's instructions (Clontech Laboratories, Inc., Mountain View, CA, USA). The lentivirus produced in this way was introduced into hfNSCs by adding the concentrated supernatants to the culture medium.

### Quantitative reverse transcription PCR

Total RNA was isolated from hfNSCs that had been cultured with a combination of FAD and S2101. Reverse transcription was performed with SuperScript VILO reverse transcriptase (Invitrogen, Waltham, MA, USA), and real‐time PCR was performed with KAPA SYBR Fast (KAPA Biosystems, Inc., Wilmington, MA, USA). The primers used are described in Table S1.

### Nuclear extract and western blot analysis

One day after treatment with FAD, the hfNSCs were washed with cold PBS and hypotonic buffer (20 mm Tris/HCl pH 7.4, 10 mm NaCl, 1.5 mm MgCl_2_) added. The nuclear extract was retrieved by centrifugation (800 ***g***). Pellet samples (nuclei) and supernatant (without nuclei; almost purely cytoplasm) for immunoblotting were prepared as follows. Pellets were lysed with lysis buffer (50 mm Tris/HCl pH 7.4, 150 mm NaCl, 1% Triton X‐100) that included a protease inhibitor cocktail (Nacalai Tesque, Inc.) and a phosphatase inhibitor cocktail (Nacalai Tesque, Inc.) for 30 min on ice. Then, sample buffer (Nacalai Tesque, Inc.) was added to the lysed samples, and the samples were incubated for 5 min at 95 °C. Aliquots of total cell lysate were separated by 5–20% SuperSep (Wako Pure Chemical Industries, Ltd., Osaka, Japan) and transferred onto polyvinylidene fluoride membranes (EMD Millipore). After blocking with BlockingOne (Nacalai Tesque, Inc.) containing 0.1% Tween 20, the membranes were incubated with appropriate primary antibodies. After washing with TBST (Tris‐buffered saline, 0.1% Tween 20), the membranes were then incubated with the appropriate HRP‐conjugated secondary antibodies (Abcam), washed, and developed with ECL Prime reagents (GE Healthcare Bio‐Sciences, Pittsburgh, PA, USA).

### Statistical analysis

We performed statistical analysis with an unpaired two‐tailed Student's *t*‐test for single comparisons. For the rescue experiments shown in Fig. 3, we used repeated‐measures ANOVA with Tukey–Kramer multiple comparison test at each point. *P *<* *0.05 was considered significant.

## Results

### Repletion of FAD promotes neuronal differentiation of hNSCs derived from the fetal cortex and iPSCs

**Figure 1:**
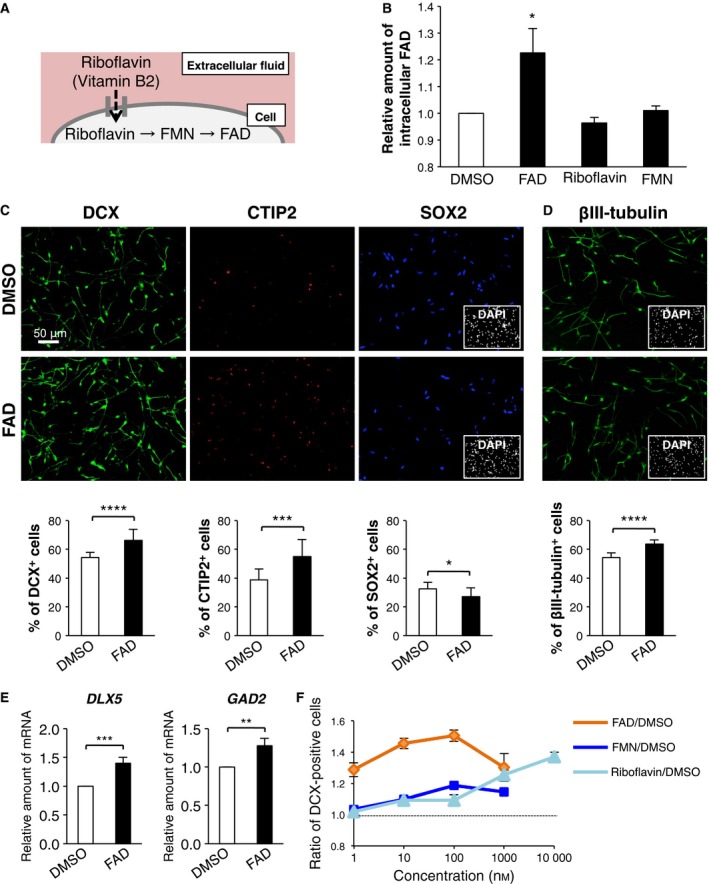
Replenishing FAD promotes neuronal differentiation of hfNSCs. (A) Overview of FAD synthesis. (B) Amount of intracellular FAD of hfNSCs treated with 100 nm FAD, 10 μm riboflavin, and 100 nm FMN for 24 h in the medium for neuronal differentiation. The values shown are the means ± SD compared with those for DMSO treatment (*N *=* *3, *t*‐test, *P <* *0.05). (C,D) Cells treated with or without FAD were stained with antibodies against DCX (green in C), CTIP2 (red in C), SOX2 (blue in C), and βIII‐tubulin (green in D) at 7 days after induction of differentiation. Insets: Hoechst nuclear staining of each field. Scale bar: 50 μm. The graphs indicate the percentage of total cells that were DCX‐, CTIP2‐, SOX2‐, and βIII‐tubulin‐positive cells. The values shown are the means ± SD. (*N *=* *6, *t*‐test, *P <* *0.05, ***P <* *0.005, ****P < 0.001). (E) Quantitative reverse transcription PCR analysis of *DLX5* and *GAD2* at 9 days after induction of differentiation. The values shown are the means ± SD compared with those for DMSO treatment. (*N *=* *3, *t*‐test, **P <* *0.01, ***P <* *0.005). (F) The graph indicates the ratio of DCX‐positive cells as compared with control cells in FAD‐, FMN‐, or riboflavin‐treated cells at 7 days after the induction of differentiation. The values shown are the means ± SD. (*N *=* *5–7).

In the cell, FAD is synthesized by several synthases, via FMN, from riboflavin that is brought into the cell by its transporter (Fig. 1A). As there is no FAD transporter present on the plasma membrane, exogenous FAD cannot be translocated into the cell under physiological conditions even though *in vivo*. Thus, to investigate the role of FAD, we hypothesized that efficient introduction of exogenous FAD into the cell would be important. Therefore, we used FAD dissolved in DMSO, which has high cell membrane permeability, in this study. Indeed, the treatment of hfNSCs with FAD dissolved in DMSO significantly for 24 h elevated the amount of intracellular FAD in the cells (Fig. 1B), in contrast to cells treated with FAD dissolved in PBS (data not shown). Furthermore, examining the absolute concentration of intracellular FAD, we found that control hfNSC was 3.83 ± 0.05 amol·cell^−1^ and that FAD‐treated hfNSC was 4.69 ± 0.35 amol·cell^−1^. Additionally, the intracellular FAD level of hfNSCs did not change after the treatment of riboflavin and FMN (Fig. 1B).

Firstly, to investigate the role of FAD in neuronal differentiation of hfNSCs, we added FAD dissolved in DMSO into the culture medium at a physiological concentration 33 for 7 days after the induction of differentiation. As shown in Fig. 1C, the FAD‐treated hfNSCs exhibited an increased percentage of doublecortin (DCX, an early neuronal marker)‐ and CTIP2 (a neuronal marker in cortical layer V/VI)‐positive cells, and a reduced percentage of SOX2 (an NSC marker)‐positive cells, in comparison with the control DMSO‐treated cells. Moreover, the number of βIII‐tubulin (an early neuronal marker)‐positive cells among the FAD‐treated hfNSCs was also increased (Fig. 1D). On the other hand, the treatment of FAD slightly reduced the number of GFAP (an astrocyte marker)‐positive cells (Fig. S1). These results indicate that the repletion of FAD in hfNSCs specifically enhances neuronal differentiation. In gene expression analysis by qRT‐PCR, we found that the inhibitory neuron marker genes, *DLX5* and *GAD2*, were upregulated after FAD treatment (Fig. 1E), suggesting that FAD influx also promoted the differentiation of hfNSCs into inhibitory neurons.

We next investigated the optimum concentration of exogenous FAD on neuronal differentiation of hfNSCs. As shown in Fig. 1F, the percentage of DCX‐positive cells was elevated in a dose‐dependent manner, peaking with a treatment of 100 nm FAD. In addition, whereas treatment with a high dose (10 μm) of riboflavin also promoted neuronal differentiation, to the same extent as FAD treatment, FMN treatment hardly had any effect, even at a high concentration (Fig. 1F). We further confirmed that FAD treatment does not induce cell death or the proliferation of hfNSCs, based on PI‐ and EdU‐uptake experiments, respectively (Fig. S2A,S2B). Taken together, these results suggest that the intracellular FAD plays an important role in the neuronal differentiation of hfNSCs.

**Figure 2:**
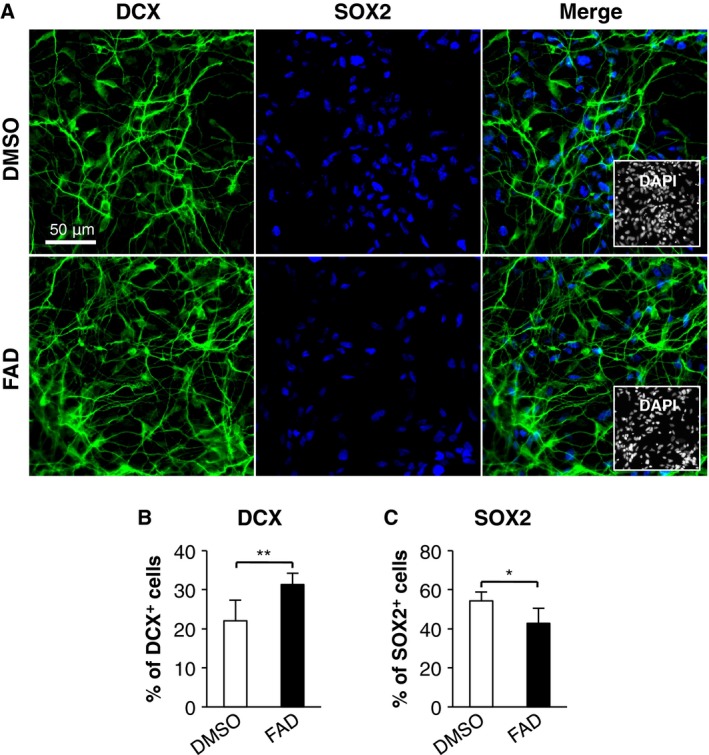
FAD promotes neuronal differentiation of hiPS‐NSCs. (A) The cells treated with or without FAD were stained with antibodies against DCX (green) and SOX2 (blue) at 10 days after induction of differentiation. Insets: Hoechst nuclear staining of each field. Scale bar: 50 μm. (B,C) The graphs indicate the percentage of total cells that were DCX‐ and SOX2‐positive. The values shown are the means ± SD. (*N *=* *6, *t*‐test, *P <* *0.05, **P <* *0.01).

Human induced pluripotent stem cell‐derived NSCs (hiPS‐NSCs) are considered to be a valuable resource for studying regenerative biology, such as studies of transplantation therapy for neurological disorders and brain injury. Thus, we considered whether the FAD treatment promotes neuronal differentiation of hiPS‐NSCs, in addition to hfNSCs. At 10 days after induction of differentiation, the percentage of DCX‐positive cells was significantly increased in the FAD‐treated hiPS‐NSCs as compared to DMSO‐treated cells (Fig. 2A,B), whereas a reduced percentage of SOX2‐positive cells was observed (Fig. 2A,C). Thus, the promotion of neuronal differentiation by FAD treatment is also applicable to hiPS‐NSCs.

### LSD1 activity is essential for FAD‐induced neuronal differentiation

**Figure 3:**
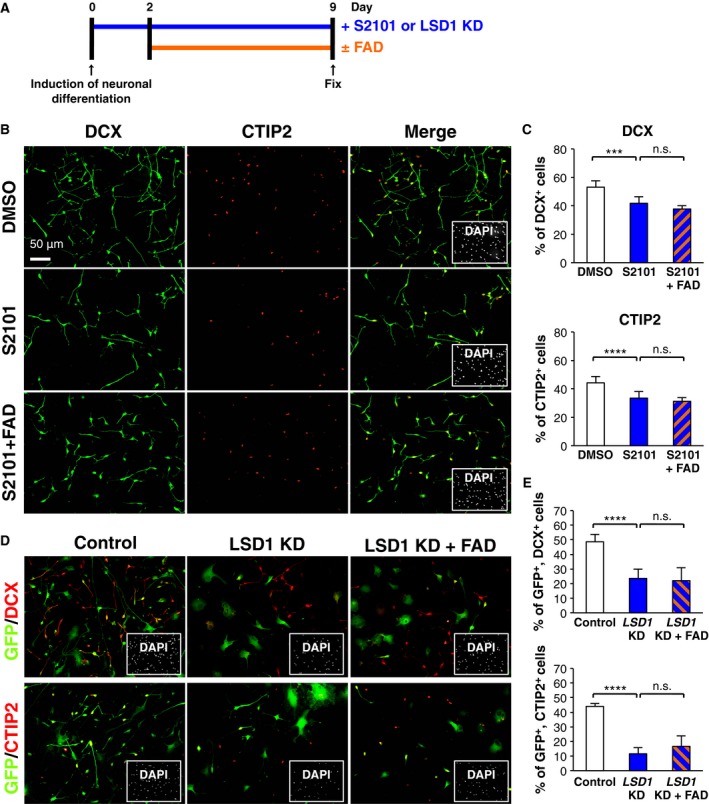
Promotion of neuronal differentiation by FAD administration mediates LSD1 activity. (A) Diagram depicting the cell culture and neuronal differentiation schedule. (B) The cells treated with S2101, FAD, or their combination were stained with antibodies against DCX (green) and CTIP2 (red) at 9 days after induction of differentiation. Insets: Hoechst nuclear staining of each field. Scale bar: 50 μm. (C) The graphs indicate the percentage of total cells that were DCX‐ and CTIP2‐positive. The values shown are the means ± SD. (*N *= 6, ANOVA, ***P < 0.005, ****P < 0.001, n.s.: not significant). (D,E) The cells, infected with recombinant lentiviruses engineered to express only GFP or GFP with shRNA designed against LSD1, were stained with antibodies against GFP (green) and DCX (red in upper panel) or CTIP2 (red in lower panel) at 7 days after induction of differentiation. The percentage of GFP‐positive cells that were DCX‐ or CTIP2‐positive was calculated. The values represent the means ± SD. (*N *=* *6, ANOVA, ****P < 0.001, n.s., not significant).

Lysine‐specific demethylase‐1 is an FAD‐dependent histone demethylase, and we recently reported that LSD1 inactivation in hfNSCs reduces neuronal differentiation by upregulating the expression of *HEYL*, one of the NOTCH target genes that has an inhibitory effect on proneuronal gene expression 26. Therefore, we hypothesized that FAD‐induced neuronal differentiation of hfNSCs would be mediated through LSD1 activation and downregulation of *HEYL*. To test this hypothesis, hfNSCs were pretreated with S2101, which is a selective LSD1 inhibitor that disturbs the association of FAD with LSD1; subsequently, cells were treated with FAD for 7 days, as shown in Fig. 3A. Consistent with our previous report, treatment with S2101 alone significantly reduced the percentages of DCX‐ and CTIP2‐positive cells (Fig. 3B,C). Notably, the percentage of these cells in hfNSCs cotreated with S2101 and FAD was similar to that of hfNSCs treated with S2101 alone (Fig. 3B,C), suggesting that binding of FAD to LSD1 is required for FAD‐induced neuronal differentiation. Moreover, differentiation of the cells into DCX‐ and CTIP2‐positive neurons was markedly suppressed in hfNSCs in which LSD1 was depressed by shRNA‐mediated knockdown, and FAD treatment of these hfNSCs did not promote differentiation of the cells into these neurons (Fig. 3D,E). This result also demonstrated that FAD‐induced neuronal differentiation is dependent on LSD1 activation in hfNSCs. Additionally, a significant increase in DCX‐positive cells at 7 days after the induction of neuronal differentiation was observed in LSD1‐overexpressing cells as compared to control cells (Fig. S3A), indicating that LSD1 activation is sufficient to induce neuronal differentiation of hfNSCs.

Given that LSD1 inactivation inhibited the neurogenesis of hfNSCs through the upregulation of *HEYL* expression in our previous study, we tested whether its expression is downregulated by FAD treatment. Although our qRT‐PCR experiment revealed statistically significantly reduced *HEYL* expression in FAD‐treated cells, the degree of reduction was small (Fig. S3B). This implies that neuronal differentiation based on LSD1 activation by FAD treatment is caused not only by the downregulation of *HEYL,* but also by the effect of other unknown target genes of LSD1.

### FAD facilitates the nuclear localization of LSD1 in hfNSCs

**Figure 4:**
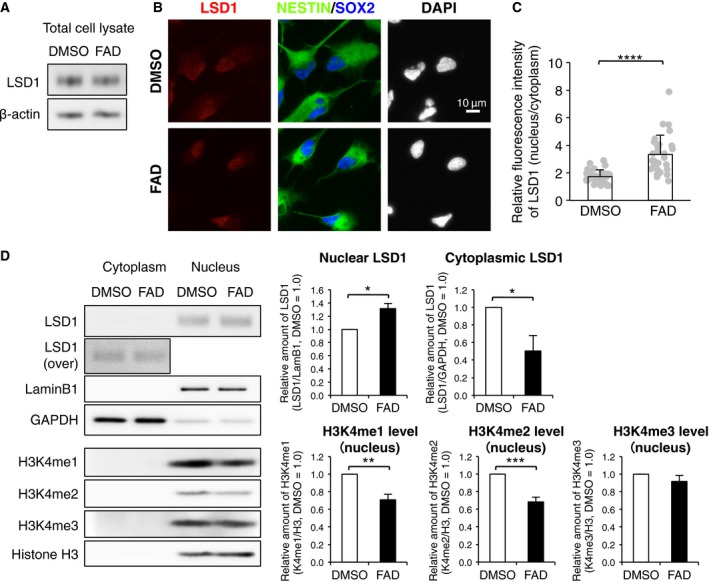
FAD facilitates the nuclear localization of LSD1 in hfNSCs. (A) Western blot analysis of LSD1 expression in FAD‐treated hfNSCs. (B) The cells treated with or without FAD for 24 h were stained with antibodies against LSD1 (red), NESTIN (green), and SOX2 (blue). Insets: Hoechst nuclear staining of each field. Scale bar: 10 μm. (C) Quantification of the ratio of fluorescence intensity of nuclear LSD1 to that of cytoplasm. The values represent the means ± SD. (*N *=* *30, *t*‐test, ****P < 0.001). (D) Western blot analysis of LSD1, H3K4me1, H3K4me2, and H3K4me3 in nuclear and cytoplasmic extracts of hfNSCs after FAD treatment for 24 h. Lamin B1 and GAPDH are used as nuclear and cytoplasmic fraction markers, respectively. In the graphs, the values of nuclear and cytoplasmic LSD1 derived from ImageStudio Digits were normalized by lamin B1 and GAPDH, respectively. The values of methylated histone were normalized by histone H3. The relative amounts are the means ± SE compared with those for DMSO treatment. (*N *=* *3, *t*‐test, *P < 0.05, **P < 0.01, ***P < 0.005).

Next, we sought to address the mechanism by which FAD influx regulates LSD1 activation in hfNSCs. Western blot analysis revealed that the LSD1 protein levels remained unchanged by FAD treatment, indicating that FAD is not involved in the expression and stability of this protein (Fig. 4A). Thus, we investigated the subcellular localization of LSD1 in hfNSCs after FAD treatment. Immunostaining showed that the signal of LSD1 immunoreactivity was broadly distributed throughout the cytoplasm and nucleus in control cells, whereas a stronger nuclear signal was detected after FAD treatment (Fig. 4B). We measured the fluorescence intensity of the LSD1 signal in the nuclear (DAPI‐positive) and cytoplasmic (DAPI‐negative) areas, and estimated the relative intensity of the nuclear signal by normalizing to the value of the cytoplasm. We found that the relative intensity of the nuclear LSD1 signal in FAD‐treated hfNSCs was significantly higher than that in control cells, suggesting that FAD influx promotes the nuclear localization of LSD1 (Fig. 4C). Supporting this, western blot analysis indicated that the amount of LSD1 protein in the nuclear fraction of FAD‐treated cells was significantly increased as compared with control cells. On the other hand, LSD1 protein in the cytoplasmic fraction displayed the opposite pattern to that of the nuclear fraction. Moreover, levels of H3K4me1 and H3K4me2 were significantly decreased by FAD treatment, whereas H3K4me3 level was not affected (Fig. 4D), indicating that FAD influx promotes the demethylation activity of LSD1.

Taking all the results of this study together, we conclude that repletion of FAD induces neuronal differentiation of human NSCs by facilitating nuclear localization of LSD1 and the demethylation activity.

## Discussion

In this study, we demonstrated that repletion of intracellular FAD promotes the neuronal differentiation of human NSCs by facilitating the nuclear localization and enzymatic activity of LSD1. Although many previous reports have indicated that intake of dietary riboflavin, a source of FAD biosynthesis, is essential for normal brain development 4, 5, 6, the details of the role of riboflavin and FAD in neuronal development have been unknown. Here, our findings not only indicate a novel role for FAD in the differentiation of somatic stem cells, but also provide a new insight that the appropriate amount of FAD derived from riboflavin in maternal nutrients is important for neurogenesis during fetal brain development.

It is well known that FAD is involved in key regulatory pathways of mitochondria, such as the metabolism of amino acids, fatty acids, and purines, and the oxidation‐reduction reaction essential for normal cellular growth and development. Recent studies have shown that neuronal differentiation of human neuronal progenitor cells requires increased ATP production through oxidative phosphorylation (OXPHOS) in mitochondria and that mitochondrial biogenesis and mitochondrial energy metabolism are elevated during differentiation of hiPS/ES‐derived NPCs into motor neurons, suggesting that an energy‐related metabolic switch underlies neuronal/glial differentiation 34, 35. In addition, Hino *et al*. 12 have demonstrated that the FAD‐dependent histone demethylase, LSD1, inhibits the transcription of genes encoding key metabolic enzymes and transcription factors, including *PGC1A* and pyruvate dehydrogenase kinase‐4 (*PDK4*). Moreover, this group also reported that LSD1 maintains glycolytic metabolism via the hypoxia‐inducible factor‐1α‐mediated pathway and reduces mitochondrial metabolism gene expression via H3K4 demethylation of their promoter regions 13. Taken together with our results, it is conceivable that H3K4 demethylation, regulated by FAD‐dependent LSD1 activation, may participate in the regulation of the energy‐related metabolic switch during neuronal differentiation of human NSCs. Further study is needed to prove this hypothesis.

We here discovered that the level of intracellular FAD mediates nuclear localization of LSD1 in hfNSCs (Fig. 4). It has been reported that the nuclear import of LSD1 is achieved by the interaction between the nuclear localization signal of the N‐terminal flexible region of LSD1 and importin α families 36. In this report, mutant LSD1 which is deleted FAD binding region can be also imported into the nucleus as well as full‐length protein 36, suggesting that binding of FAD to LSD1 does not affect on the nuclear import via importin α families. Alternatively, as recent study reported that the nuclear export of another histone demethylase, Jmjd3, is regulated by exportin‐1 37, it may be reasonable to hypothesize that FAD treatment impacts on the nuclear export of LSD1. Further understanding of how FAD regulates the nuclear localization of LSD1 is an interesting future challenge in the study.

In the present study, we show that FAD treatment induces neuronal differentiation of human NSCs derived not only from fetal brain, but also from iPSCs. Establishment of methods for efficient neuronal differentiation from human stem cells, such as NSCs, iPSCs, and ESCs, is important for disease modeling, drug screening, and cell transplantation therapy for neurodegenerative diseases. Recent advances in iPSC and ESC technology have enabled the rapid and efficient derivation of specific neurons from these stem cells by introducing proneural transcription factors and small molecules 38, 39, 40. Thus, our method of introducing FAD into cells by using DMSO might be applied for the more efficient derivation of these neurons.

## Author contributions

KH and MN involved in collection and/or assembly of data, data analysis and interpretation, manuscript writing, and conception and design.

## Supporting information


**Fig. S1.** Treatment of FAD slightly decreased the number of GFAP‐positive cells.
**Fig. S2.** Evaluation of cell death and proliferation in FAD‐treated hfNSCs.
**Fig. S3.** Overexpression of *LSD1* promotes the neuronal differentiation of hfNSCs, and the expression of *HEYL* was decreased by FAD treatment.

**Table S1**. List of gene specific primers for qRT‐PCR.
