# Supplementary figures and images for: FAD influx enhances neuronal differentiation of human neural stem cells by facilitating nuclear localization of LSD1
Source: FEBS Open Bio. 2017 Oct 17;7(12):1932–42. doi: 10.1002/2211-5463.12331 (PMC5715241; doi:10.1002/2211-5463.12331)

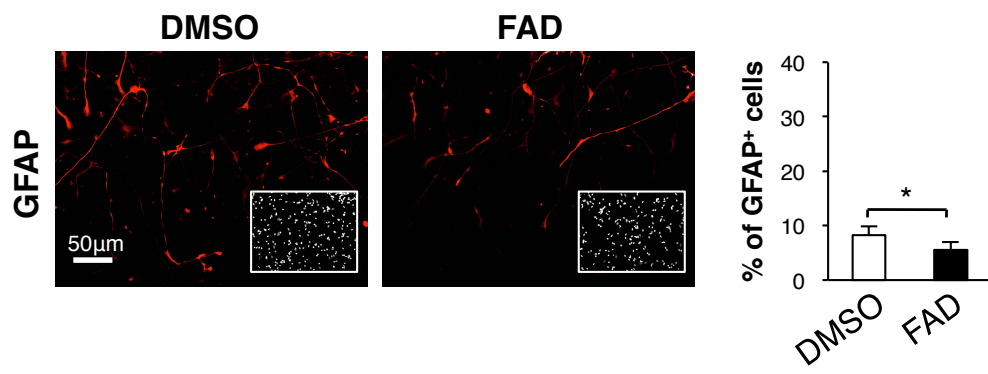

Fig. S1

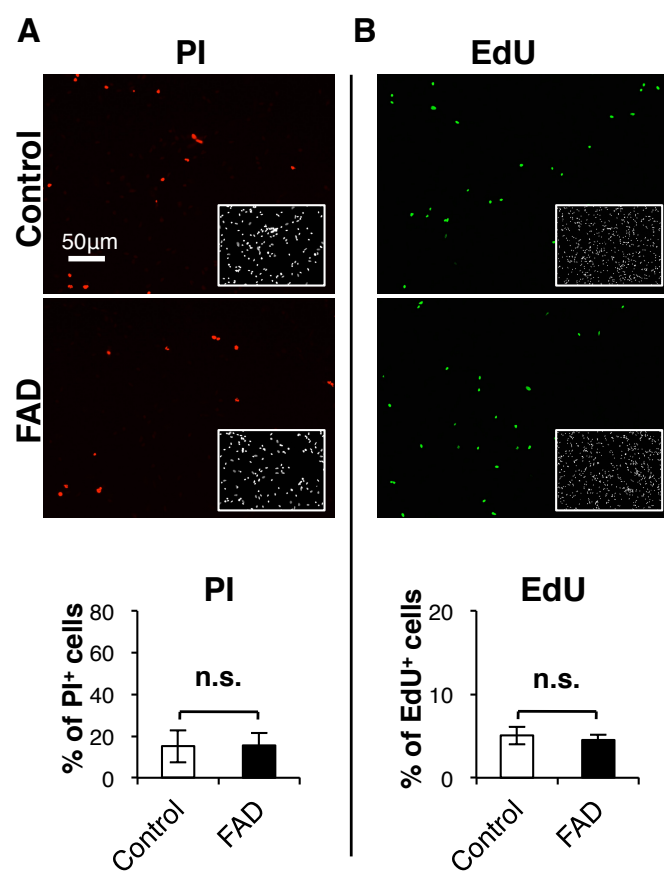

Fig. S2

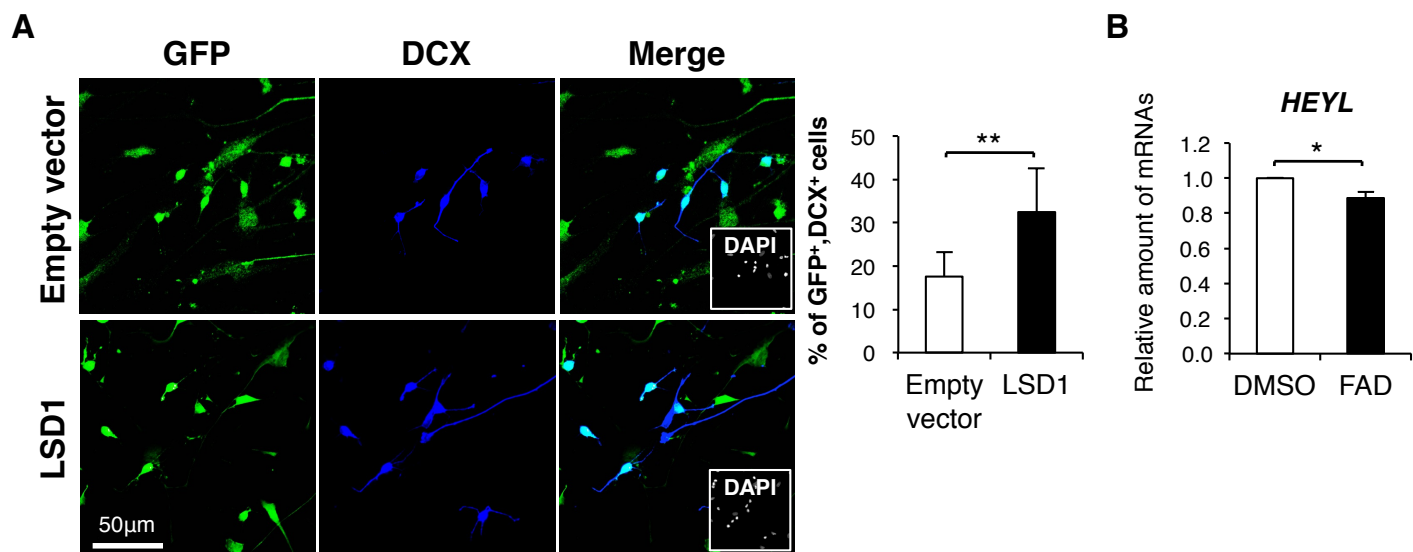

Fig. S3

Supplement: Supplementary file 1 — Fig. S1. Treatment of FAD slightly decreased the number of GFAP‐positive cells. Fig. S2. Evaluation of cell death and proliferation in FAD‐treated hfNSCs. Fig. S3. Overexpression of LSD1 promotes the neuronal differentiation of hfNSCs, and the expression of HEYL was decreased by FAD treatment. [file FEB4-7-1932-s001.pdf]
