# Supplementary material for: FAD influx enhances neuronal differentiation of human neural stem cells by facilitating nuclear localization of LSD1
Source: FEBS Open Bio. 2017 Oct 17;7(12):1932–42. doi: 10.1002/2211-5463.12331 (PMC5715241; doi:10.1002/2211-5463.12331)
Supplement: Supplementary file 2 — Table S1. List of gene specific primers for qRT‐PCR. [file FEB4-7-1932-s002.pdf]

Table S1. List of gene specific primers for qRT-PCR.

Gene	Forward primer sequence (5'-3')	Reverse primer sequence (5'-3')
hGAD2	CTGCTCCAGTCTCCAAAGCC	CCGTGAACTTCTGAGCCACT
hDLX5	ACCAACCAGCCAGAGAAAGA	GCAAGGCGAGGTACTGAGTC
h18SrRNA	GATATGCTCATGTGGTGTTG	AATCTTCTTCAGTCGCTCCA
